# Critical appraisal of the potential role of intensity modulated proton therapy in the hypofractionated treatment of advanced hepatocellular carcinoma

**DOI:** 10.1371/journal.pone.0201992

**Published:** 2018-08-13

**Authors:** Luca Cozzi, Tiziana Comito, Antonella Fogliata, Ciro Franzese, Stefano Tomatis, Marta Scorsetti

**Affiliations:** 1 Humanitas Research Hospital and Cancer Center, Radiotherapy and Radiosurgery, Milan-Rozzano, Italy; 2 Humanitas University, Dept. of Biomedical Sciences, Milan-Rozzano, Italy; North Shore Long Island Jewish Health System, UNITED STATES

## Abstract

**Purpose:**

To investigate the role of intensity modulated proton therapy (IMPT) for advanced hepatocellular carcinoma in comparison with volumetric modulated arc therapy (VMAT).

**Methods:**

An in-silico planning study was performed on 20 patients. The prescription dose was 60Gy in 6 fractions. Patients were planned with abdominal compression. IMPT plans were optimized with or without the inclusion of CT calibration (3%) and isocenter positioning (2,4,6mm) uncertainties. Plan robustness was appraised comparing rubust optimized plans vs standard plans and also in terms of the worst-case scenario. VMAT plans were optimized for 10FFF photon beams using 2 partial arcs.

**Results:**

Target coverage was fully achieved by both VMAT and IMPT plans with a significant improvement in homogeneity (~25%) with IMPT. Integral dose was reduced of ~60% with IMPT while the conformality of the dose distributions was similar among techniques. The sparing of the organs at risk was strongly improved with IMPT although all clinical objectives were met for both techniques. The inclusion of the uncertainties in the optimization lead to some deterioration in the target dose homogeneity (from 40 to 80% worse with 4 or 6mm position uncertainty) while none of the coverage parameters or OAR objective was violated. The worst-case scenario analysis demonstrated the risk of a major target underdosage only in the case of the most extreme errors (6mm) with D_98%_ in average ~12% lower than the threshold.

**Conclusion:**

IMPT with the support of abdominal compression, can be considered a viable solution also for advanced hepatocellular carcinoma patients. Great care shall be put in the minimization of the residual respiration and positioning uncertainties but the dosimetric advantage for organs at risk and the relative robustness on target coverage are promising factors.

## Introduction

Hepatocellular carcinoma is among the most common primary liver tumors and of cancer related deaths [1]. Among the various therapeutic approaches, stereotactic body radiotherapy (SBRT) demonstrated a relevant potential role [2]. Prospective and retrospective studies showed encouraging results after SBRT [3–7]. In an earlier study [8], we reported, for a cohort of 43 patients with advanced and inoperable HCC, a 64% local control at 2 years and a median overall survival of 18 months (45% survival at 2 years). Planning automation tools, like knowledge based optimization engines, [9] could streamline the otherwise complex procedure and enable a wideer application of SBRT to liver cancer patients. With a similar approach [10–12], starting from conventional fractionation and approaching moderate hypofractionation in a small cohort of 22 patients, we achieved a 95% rate of local control with a mean overall survival of 10 months. The study showed the possibility to achieve sharp dose gradients which lead to mild toxicity profiles (only 1 case of grade 3 RILD, and no other toxicities greater than grade 2).

Among the challenges inherent to the radiation treatment of liver cancer, the respiratory induced motion of the target is a matter of concern and limited the use of proton therapy. In recent years, the improvements in the delivery techniques of proton therapy, allowed to re-consider the case. Yeung [13] published a review of the status of the use of protons in the treatment of HCC concluding that proton therapy might reduce radiation-related hepatotoxicity and allow for tumor dose escalation.

Igaki [14] reviewed more in general the use of charged particles for hepatocellular carcinoma. They identified a small number of studies, inclusive of one randomized controlled trial, 9 phase I or II trials and 2 retrospective studies. The local control rates (from 71 to 95% at 3 years) and the overall survival rates (from 25 to 42% at 5 years) suggest for a positive role of the use of charged particles in general.

If, from a conceptual point of view, 4D delivery with respiratory gating should be the approach to follow, additional complication factors shall be considered with protons. In particular when spot scanning is applied, the motion mitigation techniques shall account also for the interplay of motion with the scanning patterns and, in case, the need to apply some rescanning techniques.

Zhang [15] investigated various motion mitigation strategies from the delivery point of view and concluded that the “re-gating” approach (combining rescanning with respiratory gating) could provide the most robust results for 4D plans quality, close to the static reference.

This study confirmed the earlier results of the same group [16–20] on the need of direct 4D optimization and the need of some type of rescanning technique to guarantee the adequate level of delivery quality and safety.

Poulsen [21] proposed an efficient and flexible repainting scheme, spread out over the entire breathing cycle and tested it, in-silico, for thoracic and upper abdominal patients.

A simpler approach, would be the attempt to mitigate the uncertainties due to respiration (impacting on both range uncertainty and positioning uncertainty) by means of external methods. The simplest method, routinely used for photon therapy, would be the application of abdominal compression which strongly reduces the residual motion of the internal structures. If this could be associated to some robust optimization technique (i.e. incorporation of the potential trade-off of the uncertainties in the cost functions) then it might be possible to obtain adequate plans deliverable with simpler techniques.

Aim of the present study was the assessment at planning level of the role of IMPT when compared to VMAT, the state of the art approach with photons, for advanced stage HCC patients to be treated with hypofractionated regimen. In the absence of any rescanning approach, plan robustness for proton plans was appraised and quantified to determine the potential trade-off induced by the combination of possible calibration and isocenter positioning uncertainties.

## Materials and methods

### Patients selection and dose prescription

Twenty patients affected by advanced stage HCC and unsuitable to other loco-regional therapies, were selected for this in-silico planning study. These patients were previously treated patients as described in [8]. The Humanitas Cancer Center Ethics committee approved by notification this retrospective study. All patients signed informed consent to have data from their medical records used in research at hospital admission. The study was performed by means of the analysis of the electronic records in the clinical folder and by executing dedicated planning investigations. All data were fully anonymized prior to the access.

Patients were immobilized with a thermoplastic body mask including a Styrofoam block for abdominal compression to minimize respiratory organ motion. In all patients the planning 4D-CT images were co-registered with magnetic resonance imaging (MRI) to better identify the gross tumor volume (GTV). The clinical target volume (CTV) was defined as equal to the GTV. The planning target volume (PTV) was generated from the GTV by adding an overall isotropic margin of 7mm in the cranial–caudal axis and 4–6mm in the anterior–posterior and lateral axes.

The dose prescription was of 60Gy in 6 fractions of 10Gy normalized to the mean dose to the PTV. The plan objective was to cover at least 98% of CTV volume with 98% of the prescribed dose (D_98%_ = 98%) and for PTV to cover 95% of the volume with 95% of the dose (V_95%_ = 95%). The planning objectives set for the various organs at risk for the study are reported in Table 1.

**Table 1:** Summary of the quantitative analysis of the dose volume histograms for the main structures over the entire cohort of patients for the RapidArc based photon plans and for the intensity modulated proton plans.

OARs	Objective	RA	IMPT	IMPT-arc	P
**CTV** Volume: 105±72 [33–306] cm^3^					
D_mean_	60Gy	60.0	60.0	60.0	**-**
D_2%_ [Gy]	Minimize	61.7±0.6	61.3±0.4	61.0±0.5	a,b,c
D_98%_ [Gy]	≥58.8Gy (98%)	58.8±0.4	59.0±0.3	59.2±0.4	a,b,c
V_95%_ [%]	Maximise	99.9±0.1	99.9±0.1	99.9±0.2	**-**
HI [%]	<5%	3.3±0.1	2.5±0.1	2.0±0.1	a,b,c
**PTV** Volume: 228±125 [87–578] cm^3^					
D_mean_	60Gy	59.9±0.0	60.0±0.0	60.0±0.0	**-**
D_2%_ [Gy]	Minimize	62.2±0.5	61.6±0.3	61.3±0.4	a,b,c
D_98%_ [Gy]	Maximize	56.7±1.0	57.4±0.8	57.6±0.9	a,b
V_95%_ [%]	>95%	97.4±1.5	98.5±1.0	98.6±0.9	a,b
HI [%]	<10%	6.1±0.1	4.7±0.1	4.0±0.1	a,b,c
**Healthy tissue** Volume: 35830±11314 [17030–56305] cm^3^					
V_10Gy_ [%]	-	5.8±2.6	2.5±1.2	2.3±1.0	a,b,c
CI_95%_	-	1.1±0.1	1.2±0.1	1.2±0.1	a,b,c
Dose Integral [Gy∙ cm^3^ ∙10^4^]	-	7.5±2.9	2.8±1.2	2.7±1.1	
**Liver-PTV** Volume: 1210±266 [725–1672] cm^3^					
V_<21Gy_ [cm^3^]	≥ 700 cm^3^	965±120	1093±98	1131±101	a,b
**Right kidney** Volume: 157±62 [33–241] cm^3^					
D_mean_ [Gy]	< 12 Gy	2.6±1.8	0.4±0.4	0.5±0.5	a,b
D_65%_ [Gy]	<15 Gy	0.8±0.5	0.1±0.1	0.1±0.1	a,b
**Right Lung:** Volume: 1598±315 [1053–2280] cm^3^					
D_mean_ [Gy]	<8 Gy	4.6±3.1	2.9±2.6	3.0±2.4	a,b
V_20%_ [%]	<15%	7.2±6.2	5.5±5.0	5.8±4.9	a,b
**Spinal cord** Volume: 34±16 [12–76] cm^3^					
D_1%_ [Gy]	< 27 Gy	9.4±2.1	0.6±1.3	0.4±0.6	a,b
**Stomach** Volume: 163±58 [87–299] cm^3^					
D_1%_ [Gy]	< 36 Gy	14.1±11.8	5.6±14.5	5.4±13.8	a,b
**Duodenum** Volume: 69±41 [16–155] cm^3^					
D_1%_ [Gy]	< 36 Gy	6.4±9.8	5.6±12.3	5.2±11.7	b
**Bowel bag** Volume: 1161±659 [417–2833] cm^3^					
D_mean_ [Gy]	Minimize	1.8±2.0	0.4±0.8	0.3±0.6	a,b
D_1%_ [Gy]	< 36 Gy	10.6±13.2	6.5±13.2	6.3±11.4	a,b
**Heart** Volume: ± [–] cm^3^					
D_mean_ [Gy]	<5 Gy	3.5±2.4	0.9±1.1	0.7±0.9	a,b
D_1%_ [Gy]	< 35 Gy	19.1±14.8	17.5±20.2	14.5±19.3	c

RA = RapidArc, IMPT = intensity modulated proton therapy; D_x_ = dose received by x% or xcm^3^ of the volume. D_mean_ = mean dose, V<_21Gy_ = volume receiving less then 21Gy. HI = conformity index. Statistical significance (p) a = RA vs IMPT; b = RA vs IMPT-arc; c = IMPT vs IMPT-arc

### Photon planning

Volumetric modulated arc therapy in the Rapidarc (RA) form was applied to all patients. Plans were designed for a TrueBeam linear accelerator (Varian Medical Systems, Palo Alto, USA) using 10MV flattening filter free beams with a maximum nominal dose rate of 2400 MU/min. Optimization was performed using the Photon Optimizer algorithm (v.15.07) implemented in the Eclipse planning system. Two partial arcs were chosen as the class solution for all patients. In general about 180° of gantry rotation per arc were chosen and the collimator was set in the range of 10–20° for one arc and in the range of 80–90° for the second arc. The final dose calculation was performed by means of the Acuros-XB (v.15.07) engine with a grid of 2.5mm [22].

### Proton planning

Intensity modulated proton therapy (IMPT) plans were created using beam spot scanning. The ProBeam proton system (Varian Medical systems, Palo Alto, USA) was used as a source of beam data. The fluence-based nonlinear universal Proton Optimizer (NUPO, v15.07) was used for the scope [23–25]. For the final dose calculation, the Proton Convolution Superposition algorithm (v15.07) was used. A constant RBE of 1.1 was applied. The dose calculation for all proton plans was performed on a 2.5x2.5x2.5mm^3^ grid. The spot spacing in the scanning direction was set to 5mm. Circular axial margins of 6mm from the PTV were applied as well as in the proximal and distal directions. This margin was defined coherently with the assumption of the worst-case scenario of an uncertainty in the target position (due to residual breathing or positioning errors).

Two sets of plans were computed for each case. The standard approach (IMPT) consisted in the use of a minimal number of fields. The beam arrangement chosen included two lateral-oblique fields with an eventual additional third field for the biggest targets. The gantry angles were individually determined case by case to minimise the entrance through healthy tissues and minimize or avoid the direct abutting against the right lung.

The second set of plans was optimized with the use of 10 fixed fields uniformly distributed over 360° (labelled IMPT_arc). These plans, although with a crude approximation, should mimick a proton rotational technique similar to the VMAT approach. These plans aimed to explore the potential benefit in terms of target coverage and OAR sparing of a rotational approach also for protons.

### Proton Dose Perturbation (PDP) and statistical analyses

To assess the robustness of the proton plans, PDP analysis was performed at plan level by means of two perturbation features. PDP were simulated as resulting from CT calibration errors by varying the Hounsfield Units (HU) to stopping power calibration by applying a ±3% variation (curve error). This primarily mimics the range uncertainty of the proton penetration into the patient tissues. A positive value implies a deeper penetration. The second PDP was introduced by applying a positional uncertainty in the isocentre localization. This was realised with a 4mm or 6mm shift of the isocentre along the three cartesian cohordinates. The choice of 4 and 6mm was done to account for some intermediate or extreme impact of residual breathing or positioning errors with the assumption that the treatment accuracy should be better. These CT curve calibration and positional uncertainties were then imposed during the optimization process by associating them to the CTV and OARs objectives. The optimization is then run in order to result in the solution which minimizes the trade-offs due to the applied PDPs to the dose-volume constraints in the cost function. In this sense it generates a “robust” optimization. Two sets of robust plans were optimized and calculated per each patient and labelled p_4mm and p_6mm (always including the 3% CT range uncertainty).

Finally, since also the robust optimization cannot completely eliminate the effect of the perturbations, for both the p_4mm and p_6mm plans, the final dose calculation was repeated by applying the simultaneous combination of the CT range uncertainty and the setup error on one axis at the time. This resulted in a set of additional 12 plans per group. These were labelled as RU_Δ plans (with, for example, Δ = +3%,x+4mm). The analysis of the impact of these RU_ Δ plans was limited to the worst-case scenario (i.e. the RU_ Δ plan resulting in the worse DVH) for all structures. The results will be presented in detail only for the CTV and for the liver-PTV (the liver no belonging to PTV), where the effects could be macroscopic and of clinical relevance.

### Quantitative assessment

Quantitative metrics derived from the dose volume histograms (DVH). For each structure the mean dose and a number of V_x_ and D_x_ parameters were extracted. V_x_ represents the volume receiving at least or at most an x level of dose and D_x_ the dose received by an x fraction of volume. All parameters could be expressed either in absolute (Gy or cm^3^) or relative (%) terms. For the target, the homogeneity index (HI) was scored to measure the variance of the dose. HI was defined as HI = (D_5%_-D_95%_)/D_mean_. The dose conformality was scored with the Conformity Index, CI95%, defined as the ratio between the patient volume receiving at least 95% of the prescribed dose and the PTV.

The integral dose, "DoseInt" was defined as the integral of the absorbed dose extended to over all voxels excluding those within the target volume (DoseInt dimensions are Gy*cm^3^).

The average DVH were computed, for each structure and each cohort, with a dose binning resolution of 0.02Gy.

The Wilcoxon matched-paired signed-rank test was applied to evaluate the significance of the observed differences. The threshold for statistical significance was set at <0.05.

## Results

### Dosimetric comparison:

**Fig 1:**
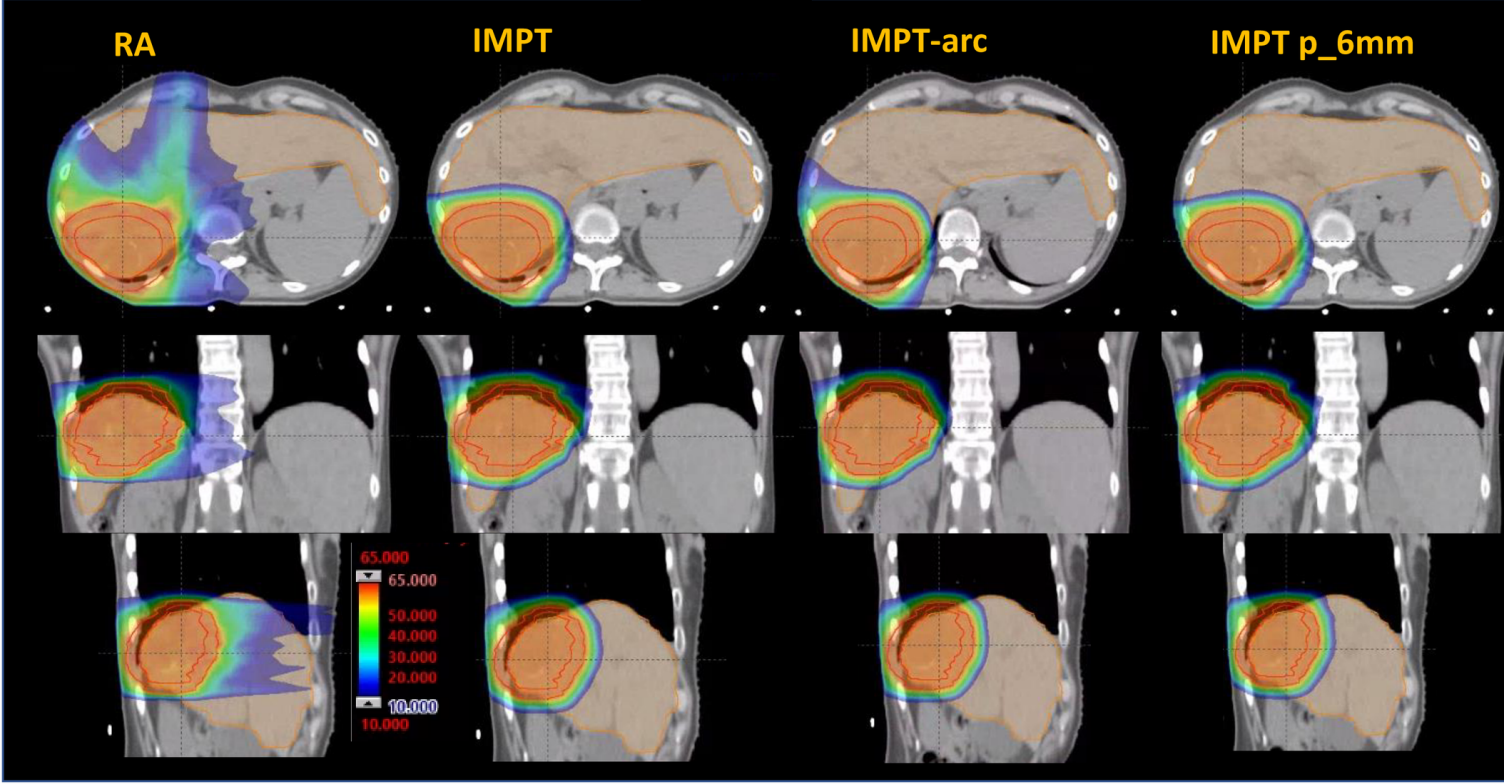
Dose distributions for an axial, coronal and sagittal plane for an example patient. The data are shown for the photon RapidArc (RA) plan and and for the proton intensity modulated plans (IMPT) as well as for the surrogate of the rotational proton plans (IMPT arc).

Fig 1 shows the dose distributions for an axial, coronal and sagittal planes for an example patient. The data are shown for the RA, for the IMPT and for the IMPT_arc. The isodose distribution is shown also for the plan obtained with robust optimization with the enforcement of the 3% and 6mm perturbations (IMPT p_6mm). The qualitative comparison illustrates well the substantial difference between protons and photons in the sparing of the organs at risk while similar coverage and conformality could be inferred.

**Fig 2:**
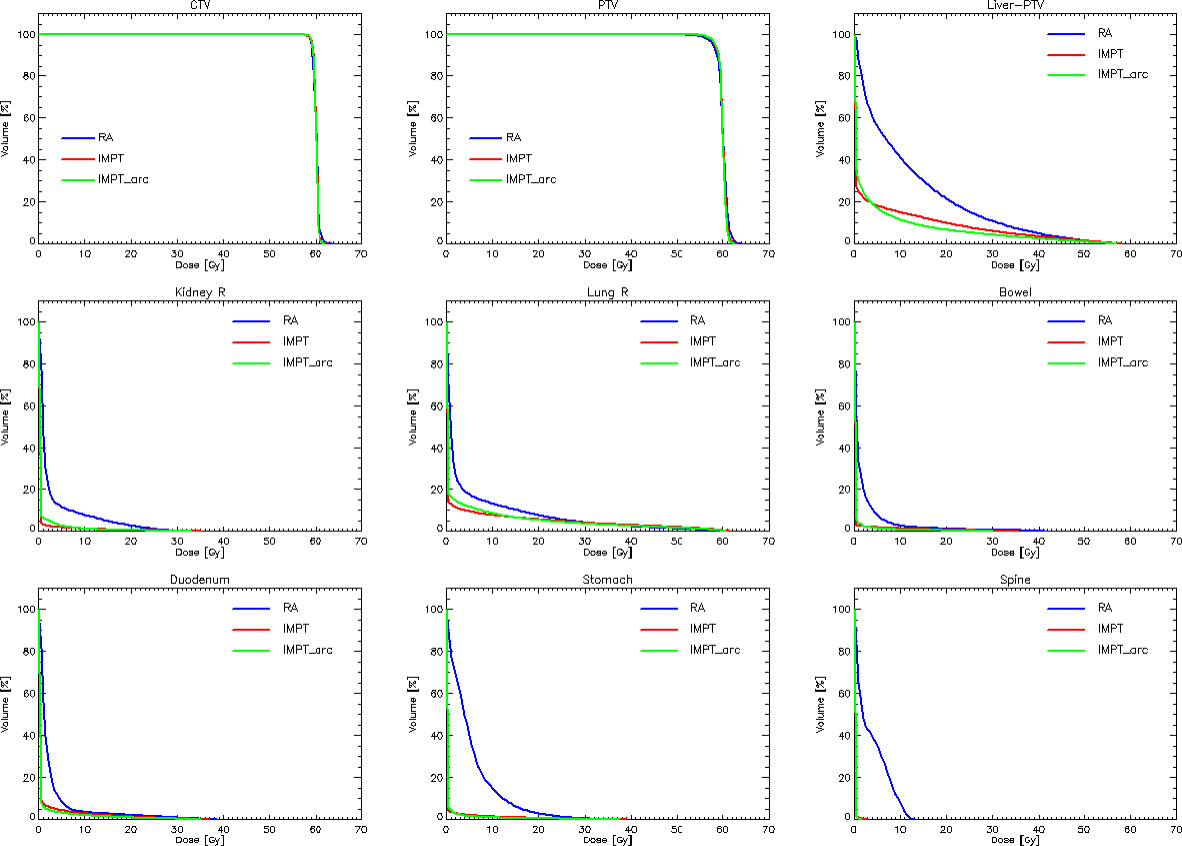
Average dose volume histograms for the CTV, PTV and the main organs at risk investigated. Data are shown for the photon RapidArc plans and for the proton intensity modulated plans (IMPT) as well as for the surrogate of the rotational proton plans (IMPT arc).

Fig 2 shows the average dose volume histograms for the CTV, PTV and the main organs at risk investigated. Data are shown for RA, IMPT and IMPT_arc plans. The graphs allow a first quantitative appraisal of the major sparing of the OARs achieved with the protons in the medium to low dose domains, irrespective from the distance of the OAR from the target volumes. The rotational approach for protons is not suggestive of significant benefits in most of the OARs with some potential positive impact onto the liver-PTV. Also the near-to-maximum doses are on average significantly impacted by the techniques and beam types.

Table 1 is the summary of the quantitative analysis of the DVHs for the three sets of plans. Data are reported as averages over the cohort of patients, and the interpatient variability is represented by the standard deviation.

Concerning the target coverage, all the planning aims were fulfilled on average for the CTV and the PTV. The dose homogeneity was roughly improved by a factor 2 for the CTV compared to the PTV. The HI for protons resulted ~25–33% better than photons for the CTV and about 23–35% for the PTV. The dose conformity resulted comparable for all the groups. In general the simulated arc proton plans improved the homogeneity of the dose with respect to the standard protons.

Concerning the global healthy tissue, the use of protons resulted in the expected macroscopic reduction of the dose bath. V_10Gy_ was reduced by a factor 2.3–2.5, while the integral dose by a factor 2.7–2.8 with respect to RA.

The portion of healthy liver receiving less then 21Gy, necessary to preserve functionality, was largely greater than the threshold for all techniques, and improved of 13–17% with the protons (better for the pseudo arc solution). For all the other OARs listed in the table, all the planning aims were met by the techniques and, as expected, the quantitative differential improvement of the protons is large. In these cases, no relevant differences were observed between the IMPT and the IMPT_arc solutions.

### On the robustness of proton plans

**Fig 3:**
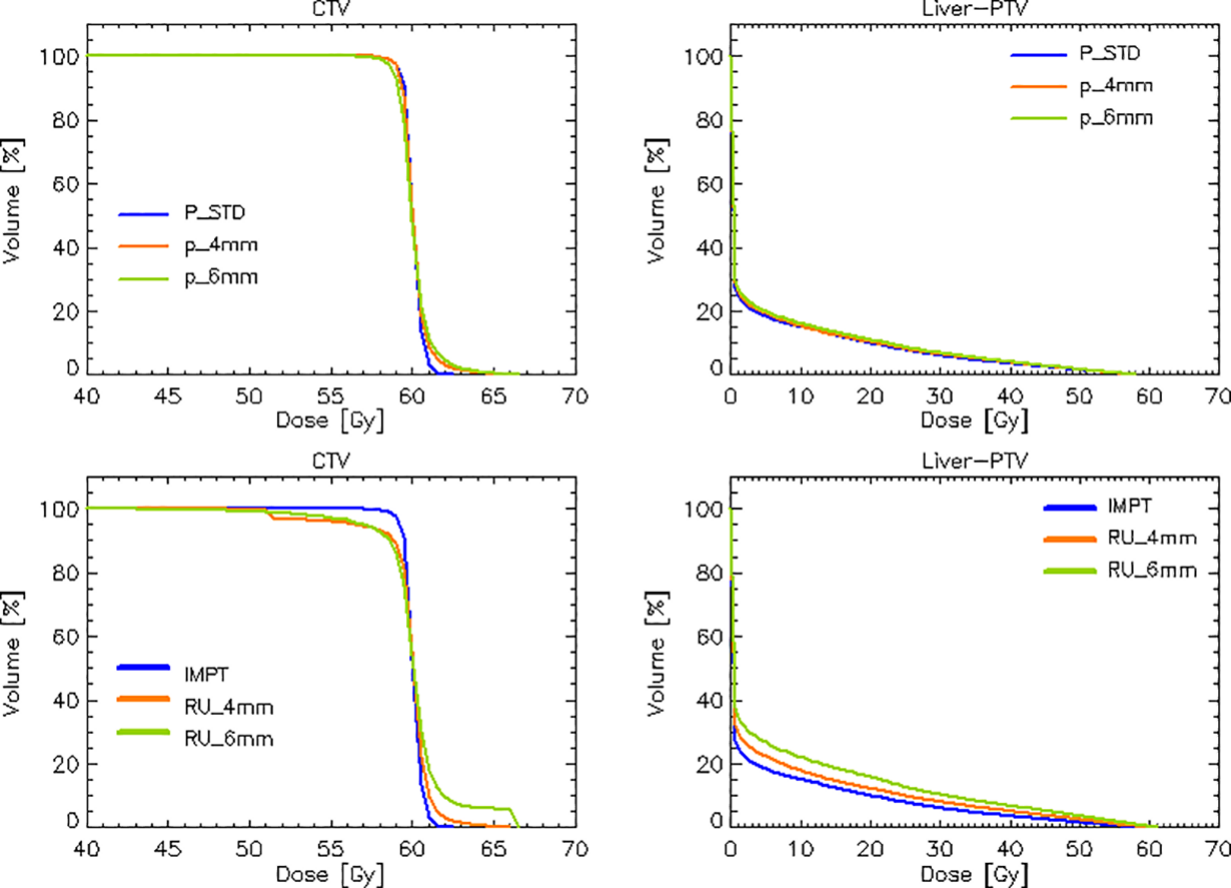
Upper row: Comparison of the average dose volume histograms for the CTV and the Liver-PTV structures for the reference IMPT plan and the two replicae obtained with the enforcement of the calibration perturbation of 3% and the positioning perturbation of 4 or 6mm. Lower row: the worst-case scenario comparing the reference IMPT plan with the plans subject to the maximal detrimental effect of each of the perturbations.

The study of the plan robustness was articulated in two phases. Firstly, the perturbations on CT calibration and isocenter position were enforced during the optimization process to generate robust plans. The results are shown, in terms of average DVH in the upper row of Fig 3 for the CTV and the Liver-PTV (the two most relevant structures and where the effects could be more relevant).

As it can be seen, the CTV showed some deterioration in the homogeneity and coverage while the average DVH for the Liver-PTV did not outline remarkable effects. Table 2 reports for the quantitative analysis for the various metrics (as in Table 1) for the IMPT, IMPT p_4mm and IMPT p_6mm plans.

**Table 2:** Summary of the quantitative analysis of the dose volume histograms for the main structures over the entire cohort of patients for the reference intensity modulated proton plan and for the two variants with robust optimization to compensate for the ±3% CT calibration error and the 4 or 6mm positioning error.

OARs	Objective	IMPT	IMPT p_4mm	IMPT p_6mm	P
**CTV** Volume: 105±72 [33–306] cm^3^					
D_mean_	60Gy	60.0	60.0	60.0	**-**
D_2%_ [Gy]	Minimize	61.3±0.4	61.8±1.5	62.2±1.6	a,b,c
D_98%_ [Gy]	≥58.8Gy (98%)	59.0±0.3	59.0±0.4	58.7±0.6	b,c
V_95%_ [%]	Maximise	99.9±0.1	99.9±0.1	99.8±0.7	
HI [%]	<5%	2.5±0.1	3.5±0.2	4.5±0.2	a,b,c
**PTV** Volume: 228±125 [87–578] cm^3^					
D_mean_	60Gy	60.0±0.0	60.0±0.0	60.0±0.0	**-**
D_2%_ [Gy]	Minimize	61.6±0.3	62.3±1.2	62.7±1.4	a,b,c
D_98%_ [Gy]	Maximize	57.4±0.8	56.8±1.8	56.5±1.8	a,b,c
V_95%_ [%]	>95%	98.5±1.0	97.2±3.5	96.6±3.8	a
HI [%]	<10%	4.7±0.2	6.0±0.3	7.1±0.3	a,b,c
**Healthy tissue** Volume: 35830±11314 [17030–56305] cm^3^					
V_10Gy_ [%]	-	2.5±1.2	2.2±1.1	2.3±1.1	-
CI_95%_	-	1.2±0.1	1.2±0.1	1.2±0.1	-
Dose Integral [Gy∙ cm^3^ ∙10^5^]	-	2.8±1.2	2.9±1.2	2.9±1.2	-
**Liver-PTV** Volume: 1210±266 [725–1672] cm^3^					
V_<21Gy_ [cm^3^]	≥ 700 cm^3^	1093±98	1089±105	1081±103	a,b,c
**Right kidney** Volume: 157±62 [33–241] cm^3^					
D_mean_ [Gy]	< 12 Gy	0.4±0.4	0.4±0.8	0.4±0.9	-
D_65%_ [Gy]	<15 Gy	<0.1	<0.1	<0.1	-
**Right Lung:** Volume: 1598±315 [1053–2280] cm^3^					
D_mean_ [Gy]	<8 Gy	2.9±2.6	2.9±2.5	2.9±2.4	-
V_20%_ [%]	<15%	5.5±5.0	5.4±4.8	5.3±4.6	-
**Spinal cord** Volume: 34±16 [12–76] cm^3^					
D_1%_ [Gy]	< 27 Gy	0.6±1.3	1.1±2.1	1.1±2.2	a
**Stomach** Volume: 163±58 [87–299] cm^3^					
D_1%_ [Gy]	< 36 Gy	5.6±14.5	6.0±15.3	6.3±15.9	c
**Duodenum** Volume: 69±41 [16–155] cm^3^					
D_1%_ [Gy]	< 36 Gy	5.6±12.3	6.6±13.5	7.0±13.8	-
**Bowel bag** Volume: 1161±659 [417–2833] cm^3^					
D_mean_ [Gy]	Minimize	0.4±0.8	0.4±1.0	0.4±1.0	-
D_1%_ [Gy]	< 36 Gy	6.5±13.2	7.5±15.4	8.0±16.5	-
**Heart** Volume: ± [–] cm^3^					
D_mean_ [Gy]	<5 Gy	0.9±1.1	1.1±1.2	1.1±1.2	a,b
D_1%_ [Gy]	< 35 Gy	17.5±20.2	19.2±20.3	19.2±20.8	a,b

IMPT = intensity modulated proton therapy; D_x_ = dose received by x% or xcm^3^ of the volume. D_mean_ = mean dose, V<_21Gy_ = volume receiving less then 21Gy. HI = conformity index. Statistical significance (p) a = IMPT vs IMPT p_4mm; b = IMPT vs IMPT p_6mm; c = IMPT p_4mm vs IMPT p_6mm

In all cases some deterioration was observed, increasing with the positional uncertainty but in no case this was sufficient to violate any of the planning aims.

The worst-case scenario was investigated for the CTV and the Liver-PTV. The lower row of Fig 3 illustrates this for the average DVHs of the reference IMPT plan with the plans subject to the maximal detrimental effect of each of the perturbations. Table 3 presents the details of this analysis. The negative trade-off on the CTV is increasing from the 4mm to the 6mm case for all the parameters and, in the case of IMPT RU_6mm the violation on the coverage requirement is of -7.9Gy (althought with a large interpatient variability), equal to 13% of the dose prescription.

**Table 3:** Summary of the quantitative impact of the range and position uncertainties. The comparison is among the reference IMPT plan, the robust optimised replica p_4mm and p_6mm and the worst-case scenario computed applying to the p_4mm and p_6mm plans the 3% and 4 or 6mm perturbations.

OARs	Objective	IMPT	IMPT P_4mm	IMPT RU_4mm	IMPT P_6mm	IMPT RU_6mm	P
**CTV** Volume: 105±72 [33–306] cm^3^							
D_mean_	60Gy	60.0	60.0	60.0±0.3	60.0	60.1±1.6	**-**
D_2%_ [Gy]	Minimize	61.3±0.4	61.8±1.5	65.8±4.1	62.2±1.6	65.9±9.2	a,b
D_98%_ [Gy]	≥58.8Gy (98%)	59.0±0.3	59.0±0.4	59.1±4.4	58.7±0.6	51.1±13.3	b
V_95%_ [%]	Maximise	99.9±0.1	99.9±0.1	99.5±13.2	99.8±0.7	95.1±9.5	**-**
HI [%]	<5%	2.5±0.1	3.5±0.2	5.6±0.4	4.5±0.2	7.8±0.8	a,b
**Liver-PTV** Volume: 1210±266 [725–1672] cm^3^							
V_<21Gy_ [cm^3^]	≥ 700 cm^3^	1093±98	1089±105	1066±128	1081±103	1024±146	a,b
D_mean_ [Gy]		4.8±3.4	5.0±3.3	6.0±3.6	5.2±3.5	7.5±5.5	a,b

IMPT = intensity modulated proton therapy; RU = ranger uncertainty; D_x_ = dose received by x% or xcm^3^ of the volume. D_mean_ = mean dose, V<_21Gy_ = volume receiving less then 21Gy. HI = conformity index.

Statistical significance (p) a = IMPT p_4mm vs IMPT RU_4mm; b = IMPT p_6mm vs IMPT RU_6mm

## Discussion

The primary aim of this in-silico planning study was to define the potential benefit of IMPT over VMAT for the radiotherapy treatment of advanced HCC patients. The data showed that the two approaches can guarantee full respect of all the planning objectives for both target volumes and organs at risk. IMPT has the advantage to significantly reduce the dose to the OARs which could be relevant in case of further dose escalation. In the study, to mitigate possible selection bias for the patients and to reflect the real distribution of patients, we considered a population characterized by small to medium size of the lesions (proven by the range of the CTV in Table 2) and uniformly localized in the various segments of the liver (upper towards the cupola, medial and central-lateral localizations). None of these factors reflected into remarkable relationship with plan quality. The targets localized in the upper segments were of course more sensitive to residual positioning uncertainties and might result in the less robust plans. In 2016, the results of a multi-Institutional Phase II Study [26] confirmed that high-dose hypofractionated proton beam therapy is an attractive radiation modality for large primary liver tumors. The interesting conclusion is that the difference in overall survival might be due to the impact of protons versus photons on post-treatment hepatic function. In the same year, Chang replied [27] that also advanced delivery of photon radiation through conformal techniques could achieve significant normal tissue sparing, sufficient to expect levels of tumor control and possible toxicity similar to proton techniques, and that the vast majority of the cases can likely be treated with either modality.

The second aim of the study was the appraisal of the robustness of the IMPT plans with respect to calibration and positioning uncertainties. The study investigated the problem under the assumption that no advanced mitigation mechanism is applied at delivery level. The pragmatic solution proposed is the use of an abdominal compression, a simple methodology successfully implemented for the photon SBRT treatments in our clinical practice.

Zhang [20] demonstrated that moderately moving patients showed liver motion of about 6mm while this could increase to more than 10mm in the larger motion cases. Nevertheless, these data were acquired for patients scanned in free breathing and without any motion mitigation strategy applied. With photons, it was demonstrated in [8] that only in 30% of the patients scanned in free breathing the respiratory excursion was greater than 5mm and that the use of abdominal compression could effectively mitigate this to less then 3-4mm. In addition, the current planning study was performed i) considering relative wide margins around the CTV, and ii) applying further 6mm extra “margin” in the setting of the proton beams (in axial, proximal and distal directions).

Lin [28] demonstrated that abdominal compression could play a role in the mitigation of residual motion effects. For small movements (less than 7mm) no further mitigation was needed. This confirms the validity of the approach proposed, in the case of small or modest motions.

All these elements concur to the assumption that also simple delivery approaches might be considerable for hypofractionated treatment of advanced stage HCC.

A third aim of the study was to speculatively ascertain whether “conventional” IMPT with a small number of fixed gantry portals could be improved by means of a simulated rotational approach. Rath [29], applied the concept of proton arc therapy to abdominal cancer patients in a slightly more complex way than in the present study. In fact, they simulated the arc with 48 static fields. We do not believe that for a proof-of-principle study such a resolution is mandatory, but we acknowledge this fact as a potential limitation of the arc approach we presented. Nevertheless, the dosimetric results do not suggest a real benefit from the application of a rotational approach.

## Conclusion

IMPT with the support of abdominal compression and with robust plan optimization process, can be considered a viable solution for inoperableHCC also without the application of complex rescanning procedures. Great care shall be put in the minimization of the residual respiration and positioning uncertainties but the dosimetric advantage for organs at risk and the relative robustness on target coverage are promising factors.
